# Commentary: Psychedelics and psychotherapy: Cognitive-behavioral approaches as default

**DOI:** 10.3389/fpsyg.2022.1020222

**Published:** 2022-09-30

**Authors:** John Burton, Austin Ratner, Timothy Cooper, Jeffrey Guss

**Affiliations:** ^1^Department of Psychiatry, Columbia University, New York, NY, United States; ^2^Committee on Public Information, American Psychoanalytic Association, New York, NY, United States; ^3^Department of Psychiatry, New York University, New York, NY, United States

**Keywords:** psychoanalysis, CBT, psychedelic-assisted psychotherapy, psychodynamic psychotherapies, ACT, DBT, psychedelics, psychodynamic psychiatry

## Introduction

We are writing to express our concern regarding the recently published article, “Psychedelics and Psychotherapy: Cognitive-Behavioral Approaches as Default.” We support the authors' efforts to address the issue of standards of care in the rapidly emerging field of psychedelic-assisted psychotherapy. However, we find fundamental problems with the argument that Cognitive-Behavior Therapy (CBT) is a pre-eminent choice in the practice of psychedelic therapy. Contrary to the authors' assertion, we suggest that any such effort to declare a “default” psychotherapy reveals an unscientific, even polemical, bias.

## The unscientific basis for CBT as “Default”

The authors assert CBT's scientific supremacy, but in their eagerness, they neglect scientific methods. CBT should, of course, be considered among the psychotherapeutic platforms used in psychedelic therapies, but no single platform can credibly lay claim to “default” status prior to collecting substantial supportive evidence. To date, no comparative studies of psychotherapy platforms have been conducted in the field of psychedelics. *Varying*, not restricting, the available treatment regimens is essential to compare effectiveness, but the authors recommend restricting the psychotherapeutic approach to their preferred methodology in advance of persuasive data. They justify their recommendation with the assertion that psychoanalytic psychotherapy is unscientific, while CBT carries an empirical “gold-standard” status. Both assumptions are demonstrably false.

The authors' assertion that psychoanalytic psychology is out of date is itself remarkably out of date. Their claim that psychoanalytic psychology lacks supportive evidence is itself lacking in supportive evidence. To make their case, the authors cite work over 40 years old (Eysenck and Wilson, 1973) and exhume philosophy professor Karl Popper's hoary 1920 argument that all psychoanalytic ideas are “non-falsifiable,” an argument rejected by subsequent philosophers of science, including Hempel (1965) and Grunbaum (1984). The authors continue their parochial agenda by attributing false centrality to a theory of “birth trauma,” as if that outdated, peripheral theory contaminates all ideas in mainstream psychoanalysis.

## The evidence for psychoanalysis

In the nearly half century since Eysenck, Wilson, and Beck decried the paucity of psychoanalytic research, neuroscience has validated core psychoanalytic concepts, including unconscious emotion and defense mechanisms (Solms, 2018). Copious research now exists on psychoanalytic efficacy (Gerber et al., 2011). There is robust evidence that psychoanalytic psychotherapy is as effective as CBT and probably has longer-lasting results (Steinert et al., 2017).

Within the psychedelic research literature itself, functional neuroimaging has begun to show an empirical basis for Freud's structural theory of the mind and a correlation between modern neurophysiological models of the Default Mode Network and the psychoanalytic concept of the Ego (Carhart-Harris et al., 2014). The authors dismiss Carhart-Harris's rigorous and meticulous work as a mere “claim.”

With increasing frequency, meanwhile, researchers have in recent years questioned CBT's claim to “gold-standard” status (Leichsenring and Steinert, 2017; Wampold et al., 2017). That claim deserves particular scrutiny in the new context of psychedelic psychotherapy. Psychedelics alter the contents of consciousness. CBT does not address, in theory or in practice, the dynamic relations between conscious and unconscious states. Psychoanalytic psychology, by contrast, was founded on the study of these relations. Psychedelics instigate complex changes in patients' attitudes to self and others that are conceivably best understood and supported with psychoanalytic models. Attempting to short-circuit exploration of a role for psychoanalytic psychotherapy in conjunction with psychedelics is not only unscientific, it is not in the best interest of patients.

## Discussion

Why would the emerging field of psychedelic-assisted psychotherapy rigidly limit itself to one “default” therapeutic model, except for ideological and emotional reasons? The authors' rush to claim the new territory of psychedelic-assisted psychotherapy for CBT as “default” demonstrates how such claims to “gold-standard” status sometimes do not serve a scientific agenda, but instead do a disservice to patients and practitioners alike.

We offer the above comments not to disparage CBT as a viable and effective model of treatment, but to flag the problem of activism and bias masquerading as science. Especially in the new and exciting field of psychedelic therapy, it is important not to prematurely reject valuable tools, such as psychoanalytic psychology, in order to support one particular agenda and denigrate others.

## Author contributions

JB, AR, and TC made initial contributions to the manuscript. AR wrote the first draft. JG provided subsequent contributions and collaborated with JB and AR to create the final version. All authors contributed to the article and approved the submitted version.

## Conflict of interest

The authors declare that the research was conducted in the absence of any commercial or financial relationships that could be construed as a potential conflict of interest.

## Publisher's note

All claims expressed in this article are solely those of the authors and do not necessarily represent those of their affiliated organizations, or those of the publisher, the editors and the reviewers. Any product that may be evaluated in this article, or claim that may be made by its manufacturer, is not guaranteed or endorsed by the publisher.
